# Pupillary dynamics predict long‐term outcome in a cohort of acute traumatic brain injury coma patients

**DOI:** 10.1002/acn3.51879

**Published:** 2023-08-28

**Authors:** Elena Monai, Chiara Favaretto, Anna Salvalaggio, Lorenzo Pini, Marina Munari, Maurizio Corbetta

**Affiliations:** ^1^ Clinica Neurologica University Hospital of Padova Padova Italy; ^2^ Department of Neuroscience University of Padova Padova Italy; ^3^ Padova Neuroscience Center (PNC) University of Padova Padova Italy; ^4^ Neuro‐Intensive Care Unit University Hospital of Padova Padova Italy; ^5^ Venetian Institute of Molecular Medicine (VIMM) Padova Italy

## Abstract

**Objective:**

Examining the size and reactivity of the pupils of traumatic brain injury coma patients is fundamental in the Neuro‐intensive care unit (ICU). Pupil parameters on admission predict long‐term clinical outcomes. However, little is known about the *dynamics* of pupillary parameters and their potential value for outcome prediction.

**Methods:**

This study applied a time‐course analysis of pupillary signals (size and photo‐reactivity) in acute traumatic brain injury coma patients (*n = 20*) to predict outcome at 6 months.

**Results:**

The time course of pupillary signals was informative in discriminating favorable (F) versus unfavorable (U) outcomes, with the highest correlation within the 1st week notwithstanding pharmacological sedation. Patients with favorable outcome at 6 months showed more consistent in time isochoric and photo‐reactive pupils. In contrast, patients with an unfavorable outcome showed more variable measures that tended to stabilize toward pathological values.

**Interpretation:**

Time‐dependent tracking of pupils' size and reactivity is a promising application for ICU monitoring and long‐term prognosis. These findings support the usefulness of automatic tools for the dynamic, quantitative, and objective measurements of pupils.

## Introduction

The pupillary exam is a fundamental part of the neurological evaluation of traumatic brain injury (TBI) coma patients. It represents the bedrock of the clinical evaluation of the integrity of the brainstem and arousal centers.^1^

Pupils' assessments guide clinicians to detect neurological deterioration that requires immediate treatment in unresponsive patients. Moreover, pupillary indices are used on admission as prognostic scores for long‐term prognosis in TBI coma patients.^2^, ^3^, ^4^

In the USA alone, 1.7 million people suffer from TBI, with a mortality rate of 3%^5^ and long‐term neurological sequelae that impact the quality of life.^6^ About 10–15% of acute TBI patients require management in the ICU^7^; and an accurate long‐term prognosis in the acute phase remains a challenge.^8^

The predictive scores commonly used in clinical practice measure pupils at a single time point (i.e., on admission).^3^, ^4^, ^5^ However, recent studies have highlighted the potential importance of the dynamics of bio signals in critically ill patients.^9^, ^10^, ^11^ For instance, there is a correlation between pupillary signals in the few hours preceding a raise in intracranial pressure (ICP) and the severity of increased ICP episodes and long‐term outcome in patients with severe TBI.^12^

To our knowledge, no previous study has tracked the entire time course of pupil's size and reactivity in TBI coma patients from admission to discharge from the ICU to assess its correlation with long‐term outcome as compared to a single evaluation on admission.

The aim of the present study was, therefore, to apply a time‐course analysis of pupillary signals in a group of comatose TBI patients admitted and followed in the Neuro‐intensive care unit (Neuro‐ICU) and relate pupillary dynamics (both size and photo‐reactivity) observed for an extended time‐window to outcomes assessed at 6 months post‐trauma.

## Subjects/Materials and Methods

### Patients

This retrospective study involved a cohort of acute coma patients (*n* = 21, F = 5, Age 45.5 ± 18.09 years) admitted to the Neuro‐ICU of the University Hospital of Padova after TBI over a period of 2 years (2016–2018). The study was approved by the local ethical committee at the University Hospital of Padova (n.0048695), and patients' relatives gave informed consent.

The inclusion criteria were: age > 18 years; closed brain injury; admission to the Neuro‐ICU under sedation with a Glasgow Coma Scale (GCS) ≤ 8. Exclusion criteria were: analgosedation lasting <24 h.

All patients had closed brain injury due to vehicle accidents or falls. The study population was divided into two groups according to the Glasgow Outcome Scale Extended (GOSE) at 6 months (outcome index): 15 patients had a favorable (F) prognosis (GOSE>4) and six had an unfavorable (U) prognosis (GOSE ≤ 4).

The length of sedation was 9 ± 6 days (10 ± 6 and 8 ± 6.98 days, respectively, in the F and U groups), and the length of stay in the ICU was 17 ± 9 days (18 ± 8 and 15 ± 11.72, respectively, in the F and U groups) (Table 1).

**Table 1:** Clinical data of traumatic brain injury (TBI) patients: Numbers of patients, ratio female/male, age (mean ± SD in years), findings at CT scan (contusion, traumatic subarachnoid hemorrhage [tSAH], extradural hematoma [EDH], intraventricular hemorrhage [IVH]), number of patients that underwent neurosurgery (NS), Marshall score, number of patients with invasive neuromonitoring (INM), days of sedation and ICU stay (mean ± SD) in F and U group, etiology of TBI, past medical history in the two outcome groups.

	*N*	Age	Gender (F)	Contusion	tSAH	EDH	IVH	NS	Marshall score	INM	Sedation	ICU Stay	Etiology (road accident/fall/unknown)	Past medical history
Favorable	15	43.06 ± 16.15	4	14	12	5	0	3	10 (II), 2 (III), 1 (V), 2 (VI)	10	10 ± 6	18 ± 8	7/2/06	2 HTN, 2DMII, 2 previous TBI, 1 depression
Unfavorable	6	46 ± 23.12	1	6	0	0	1	3	1 (II), 3 (III), 2 (V), 0 (VI)	5	8 ± 7	15 ± 12	3/1/02	2 HTN, 1 previous TBI and depression
*p* value (*t* statistic)		0.743 (0.333)	0.95 (0.0065)	0.62 (024)	0.004 (8.17)	0.29 (1.11)	0.62 (0.24)	0.40 (0.71)	0.06 (7.40)	0.81 (0.05)	0.65 (0.46)	0.46 (0.76)		

Sixteen patients were reported to have a GCS ≤ 8 at the trauma scene, and five patients underwent pharmacological‐induced coma.

All patients received analgosedatives (propofol, midazolam, and remifentanil; patients three and seven also received thiopental) and vasoactive drugs. All patients were intubated and under mechanical ventilation upon admission. Fifteen patients were invasively monitored to measure ICP and cerebral perfusion pressure and were treated according to current guidelines. Six patients underwent decompressive craniectomy or hematoma evacuation (patients 3, 7, 9, 14, 15, 16) (Table 1).

The CT scans of all patients were classified according to the Marshall score. Five patients (3 F and 2 U) also underwent brain MRI scans that, in all five patients, revealed diffuse axonal injury. For each patient, the type and dose of sedative drugs, as well as the length of sedation, were recorded (Table 1). Patient 12 was excluded from the pupillary analysis of the present study due to an optic nerve post‐traumatic injury.

Clinical variables were compared between the two outcome groups using a *t*‐test analysis for quantitative variables and a chi‐square for qualitative or categorical variables. The two groups were not statistically different in terms of clinical variables except for a higher prevalence of tSAH in the F group (Table 1).

Of the 21 patients initially included in the study, one patient (patient 12) was then excluded from the pupillary analysis due to the presence of post‐traumatic optic nerve injury.

### Outcome

**Figure 1:**
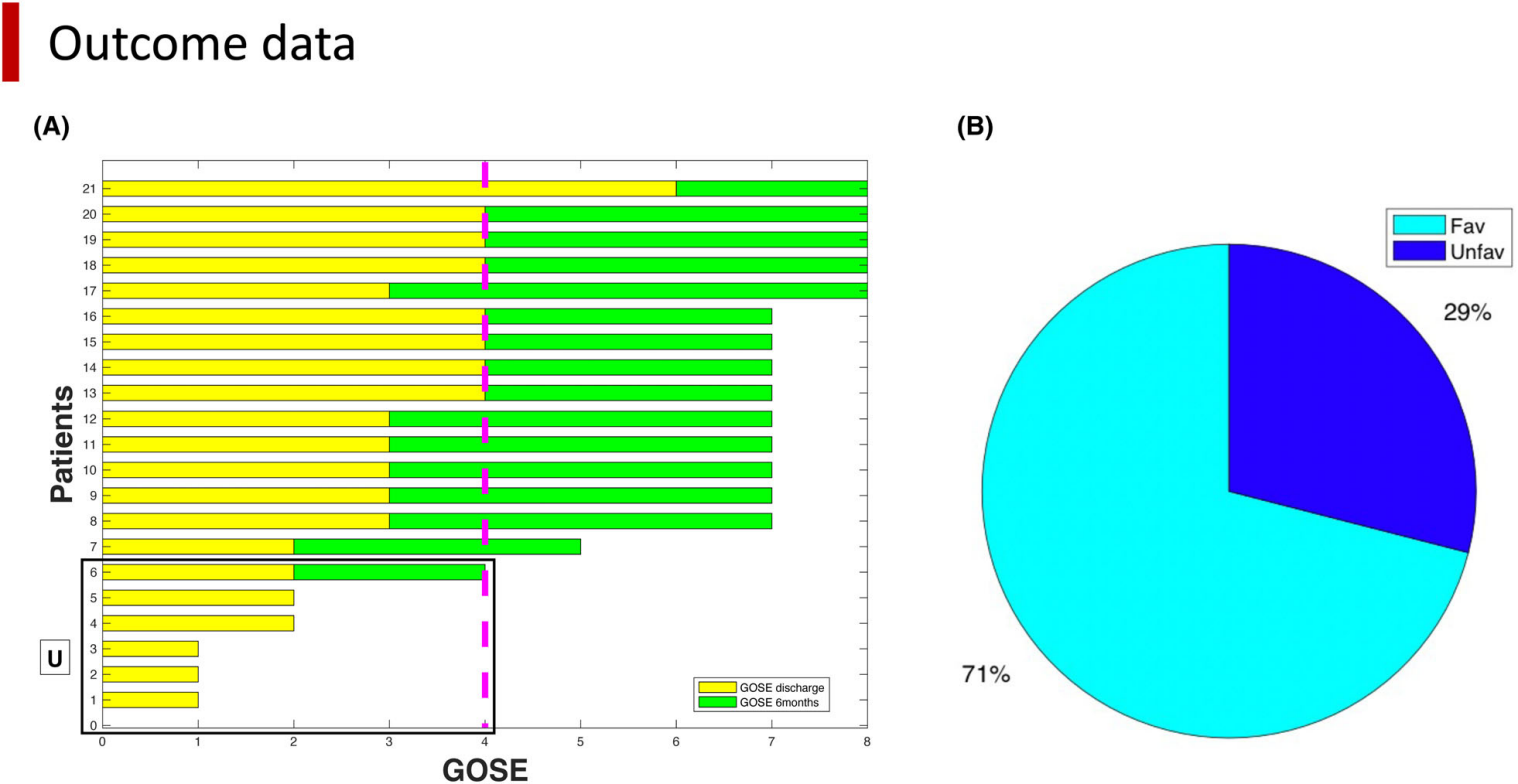
Outcome of traumatic brain injury patients: (A) GOSE values at discharge (yellow) and at 6 months (green) are displayed for each patient on the x axis. On the y axis, patients are ordered from the lowest to the highest GOSE at 6 months (1 = patient 3; 2 = patient 9; 3 = patient 10; 4 = patient 2; 5 = patient 15; 6 = patient 20; 7 = patient 14; 8–16 = patients 4, 5, 7, 8, 11, 12, 16, 17, 18; 17–21 = patients 1, 6, 14, 19, 21). (B) % of patients with favorable (fav = light blue) versus unfavorable outcome (unfav = dark blue) computed at 6 months. GOSE = Glasgow Outcome Scale Extended.

The GOSE was calculated at discharge and 6 months (Fig. 1). The GOSE at 6 months was assessed by clinicians during a follow‐up visit or through a phone interview. Outcome was dichotomized into favorable (4 < GOSE **≤** 8) versus unfavorable (1 < GOSE **≤** 4) based on GOSE at 6 months, except for patient 2, for which only the discharge’ GOSE was available.

Fifteen patients had a favorable outcome (patients 1, 4, 5, 6, 7, 8, 11, 12, 13, 14, 16, 17, 18, 19, 21) versus six patients with an unfavorable outcome (patients 2, 3, 9, 10, 15, 20) (Fig. 1).

At discharge, all patients had GOSE ≤ 4 except patient 13. GOSE improved at 6 months for all patients except patient 15 and the three patients with brain death (patients 3, 9, 10).

At 6 months in the F group, four patients had upper good recovery, nine lower good recovery, one lower moderate disability, one upper severe disability.

In the U group, three patients were in brain death (patients 3, 9, 10) while patient 15 was in a persistent vegetative state.

### Pupils

Two pupils' indices (i.e., size and photo‐reactivity) were manually assessed using a light pen every 2 h. The possible findings were as follows: (1) isochoric, (2) anisochoric (>0.5 mm difference), (3) bilaterally myotic (<2 mm); and, (4) bilaterally mydriatic (>5 mm) pupils, with the light reflex present or absent (unilaterally or bilaterally).

Therefore, four different size abnormalities were considered for the analysis (left and right anisochoria, bilateral miosis, and bilateral mydriasis). To account for the time evolution of pupil size (PS) abnormalities, we defined two variables: the PS‐jump‐rate and the PS‐isochoria‐percentage. The first index is the ratio between the number of changes of pupil condition and the number of measurements; the second index measured the percentage of time points during which the pupil size was bilaterally normal and symmetric (isochoria).

The values for pupillary light reflex were reported as bilateral presence, bilateral absence, unilateral presence (right or left). Thus, three levels of abnormality were considered. As for the pupils' size information, we defined two dynamical variables to describe photo‐reactivity: first, the ratio between the number of changes of photo‐reactivity condition and the number of measurements (PR‐jump rate); second, the percentage of time points during which the photo‐reactivity was normal (PR‐presence‐percentage).

These variables were first plotted for each patient for the whole duration of the admission in the Neuro‐ICU, except for patient 12 who suffered an optic nerve injury that prevented a normal pupillary assessment. Therefore, the pupillary analysis was performed in 20 patients (14 F/6 U).

The pupillary indices (PS‐jump‐rate, PS‐isochoria‐percentage, PR‐jump rate, and PR‐presence percentage) were independent variables in a repeated‐measured (over time) ANOVA testing for differences between F‐outcome and U‐outcome patients during the sedation and post‐sedation period.

Next, given both pupils' size and light reactivity tend to stabilize after some days during sedation, we tested whether these measures discriminate F‐ and U‐outcome patients using a different time interval from the first day of the ICU stay. Specifically, we considered the four measures (PS‐jumps rate, PS‐isochoria‐percentage, PR‐jumps rate, PR‐presence‐percentage) and evaluated them from 0 to 10 days after admission. For each number of days (NDs), we first computed the point‐biserial correlation of these measures with outcome (F vs. U); next, we estimated a logistic regression model to predict the outcome using these measures as predictors (one at a time, or combined). At each ND, we selected the best model based on three model scores, namely the model significance (p‐value), the Bayesian information criterion (BIC, the lower the better), and the accuracy level, estimated as the percentage of correct outcome prediction using a leave‐one‐out cross validation analysis.

## Results

### Time‐course of pupillary indices and outcome

**Figure 2:**
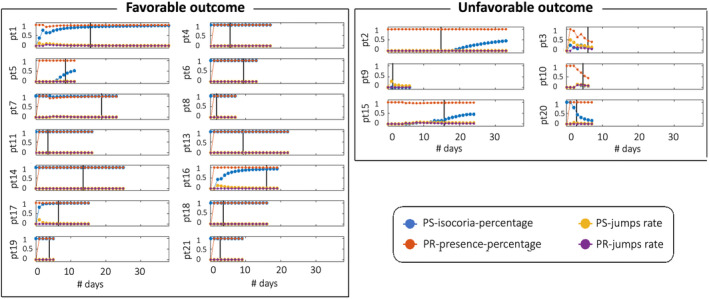
Time course of pupillary size and reactivity index. Time course of pupillary size and reactivity index (PS‐jumps‐rate; PS‐isochoria‐percentage; PR‐jumps‐rate; PR‐presence‐percentage) are plotted for each patient in the two outcome groups for the entire length of stay in the Neuro‐intensive care unit (number of days on the x axis). The black vertical lines indicate the stop of sedation. PR, photo‐reactivity; PS, pupil size.

Figure 2 shows that both pupillary size and photo‐reactivity indexes tend to stabilize over time in the F‐outcome group (e.g., patients 1, 5, 16). Instead, the U‐outcome group is characterized by a more variable temporal trend (e.g., patients 3, 10, 20) or delayed partial normalization (e.g., patients 2, 15).

Starting from these observations, we first tested whether the evaluation of these indices, even when patients are still under sedation, can be informative of patients' long‐term outcome.

**Figure 3:**
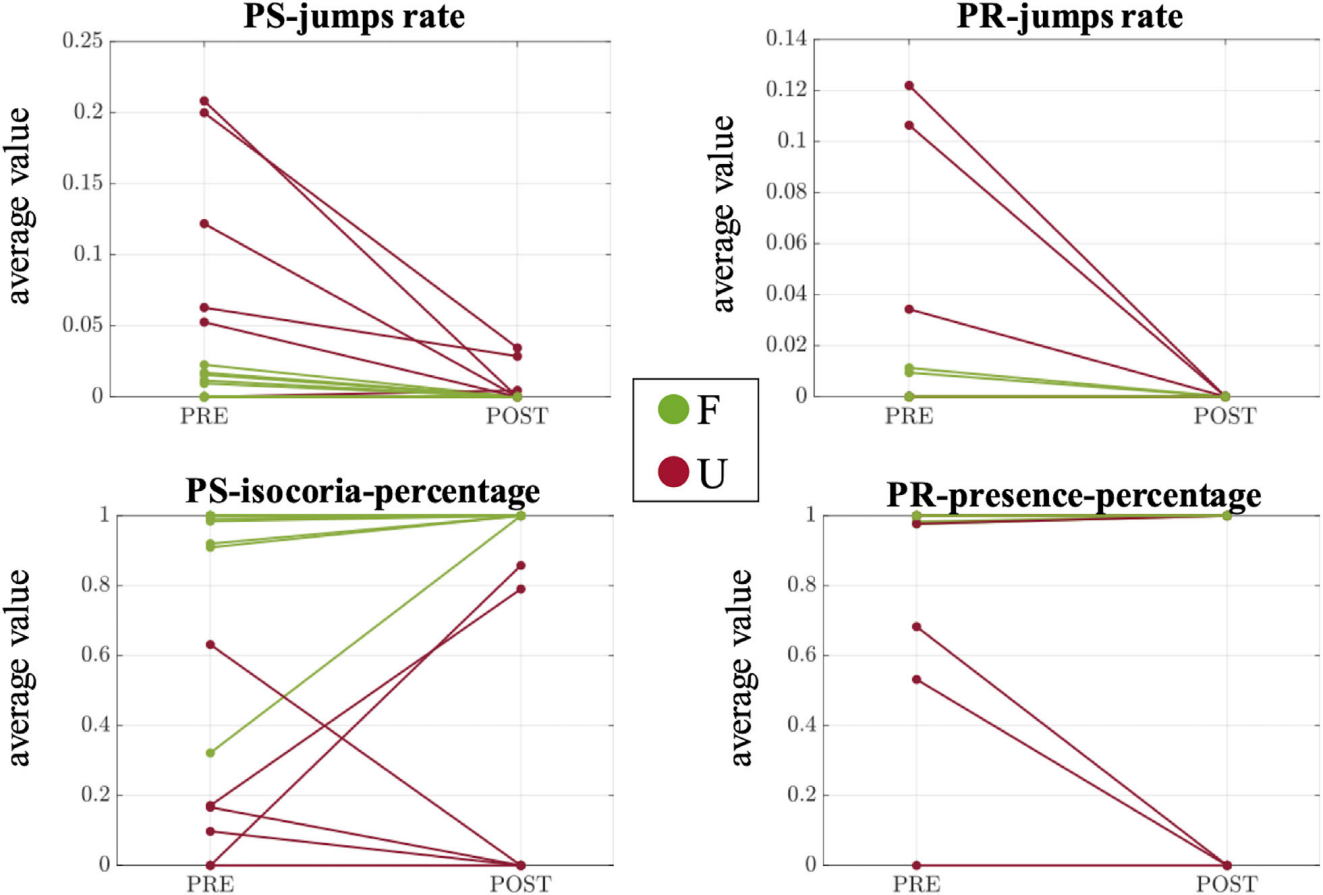
Average pupillary measures pre and post sedation. Average values of pupillary size and photo‐reactivity indexes for each patient, during sedation (PRE) and after sedation ends (POST). Favorable (F) and Unfavorable (U) outcomes are color‐coded, respectively, in green and red. PR, photo‐reactivity; PS, pupil size.

For each index (PS‐jumps‐rate; PS‐isochoria‐percentage; PR‐jumps‐rate; PR‐presence‐percentage) a repeated‐measures ANOVA with pre and post sedation periods as within factor, and outcome (F‐ vs. U‐) as between factor was run (Fig. 3). For the PS‐isochoria‐percentage measure there was no difference between pre‐ and post‐sedation, nor an interaction with outcome. However, there was a highly significant difference between outcome groups (*F* = 129.383, *p* < 0.001). Patients with F‐outcome showed longer periods of isochoric pupils. Importantly, this result is still significant when only the data under sedation is considered (*F* = 62.691, *p* < 0.001); this result indicates that this measure can discriminate F versus U‐outcome patients already during the first days of admission despite the presence of sedation.

A similar behavior was observed for PS‐ and PR‐jump rate, where a significant interaction of period (pre‐ post‐sedation) and outcome (F vs. U) was found (PS‐jump: *F* = 17.959, *p* < 0.001); PR‐jump: *F* = 9.101, *p* = 0.007). There were also significant main factors for both period and outcome (PS‐jump: pre > post: *F* = 22.526, *p* < 0.001, F < U: *F* = 24.064, *p* < 0.001; PR‐jump: pre > post: *F* = 10.339, *p* = 0.005, F < U: *F* = 9.109, *p* = 0.007). These analyses indicate two main results. First, these indices stabilize after the end of sedation with a decreased variability. Second, this variability is significantly less for patients with F‐outcome. Importantly, from a post‐hoc analysis, both jump‐rate indexes indicated a significant difference between outcomes during sedation (PS: Upre > Fpre: *t* = 6.478, *p* < 0.001; PR: Upre > Fpre: *t* = 4.268, *p* < 0.001). On the contrary, after sedation this difference was not any longer significant since both measures stabilize. Indeed, we observed a significant difference between pre and post sedation end only for the U group (PS: Upre > Upost: *t* = 5.369, *p* < 0.001; PR: Upre > Upost: *t* = 3.688, *p* = 0.009).

Finally, with regard to the PR‐presence‐percentage measure, there was both a significant period x outcome interaction (*F* = 6.427, *p* = 0.02) and significant main effects (pre > post: *F* = 6.086, *p* = 0.023; F > U: *F* = 4.664, *p* = 0.044). The post‐hoc analysis showed that the interaction reflects a significant reduction of PR‐presence‐percentage in the U group (Upre > Upost: *t* = 2.959, *p* = 0.48). All post‐hoc *p*‐values reported are Bonferroni corrected for six multiple comparisons.

Given our pupillary size and photo‐reactivity indexes discriminated between favorable and unfavorable outcomes in the course of sedation, we focused on the first day of the ICU stay to examine the optimal number of days to ascertain a significant prediction of outcome at 6 months.

**Figure 4:**
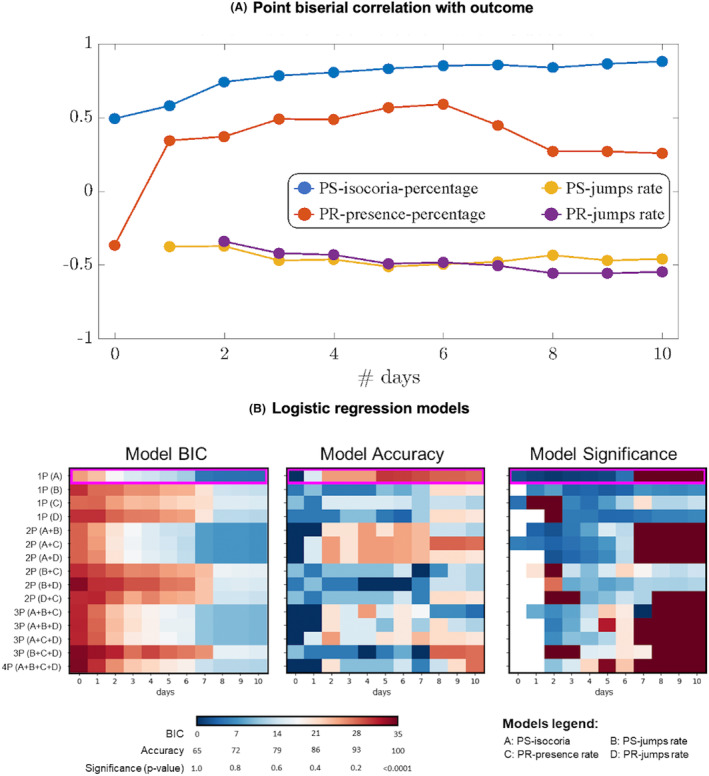
(A) Correlation between pupillary indices and outcome. Point biserial correlation between the outcome (Favorable vs. Unfavorable) and each index (PS‐jumps‐rate; PS‐isochoria‐percentage; PR‐jumps‐rate; PR‐presence‐percentage) during the first 10 days of stay in the Neuro‐intensive care unit (x axis). Filled dots indicate significant correlation values. (B) Prediction models: Results of the model selection analysis, where the outcome is considered as the dependent variable and all possible combination of the four indexes (A–D) are used as predictors. Left = Model Bayesian information criterion (the lower the better); Middle = Model accuracy (the higher the better); Right = Model significance (1 ‐ *p*‐value, the larger the better). PR, photo‐reactivity; PS, pupil size.

Figure 4A shows a point‐biserial correlation analysis between outcome and each of the four indexes. PS‐isochoria‐percentage and PR‐presence‐percentage showed a significant correlation with outcome since Day 1. Correspondingly, both PS‐ and PR‐jump‐rate indexes are significantly negatively correlated with outcome starting on Days 1–2. The PS‐isochoria‐percentage—the index with the highest absolute correlation value—increases up to 5–6 days then it stabilizes.

From a model selection analysis run among all possible predictor combinations and based on three performance indexes, namely model significance (*p*‐value), model BIC, and model accuracy (evaluated through a leave‐one–one cross‐validation analysis) (Fig. 4B), we discovered that the PS‐isochoria‐percentage index was the best measure in predicting the long‐term outcome in terms of BIC (13.56) and accuracy (95) when measured alone for the first 5 days. At Day 5, the model showed a significant *p*‐value (<0.05) when the PS‐isochoria‐percentage was combined with the other pupillary indices (i.e., all possible predictors were considered together) (Fig. 4B and Table S1).

**Figure 5:**
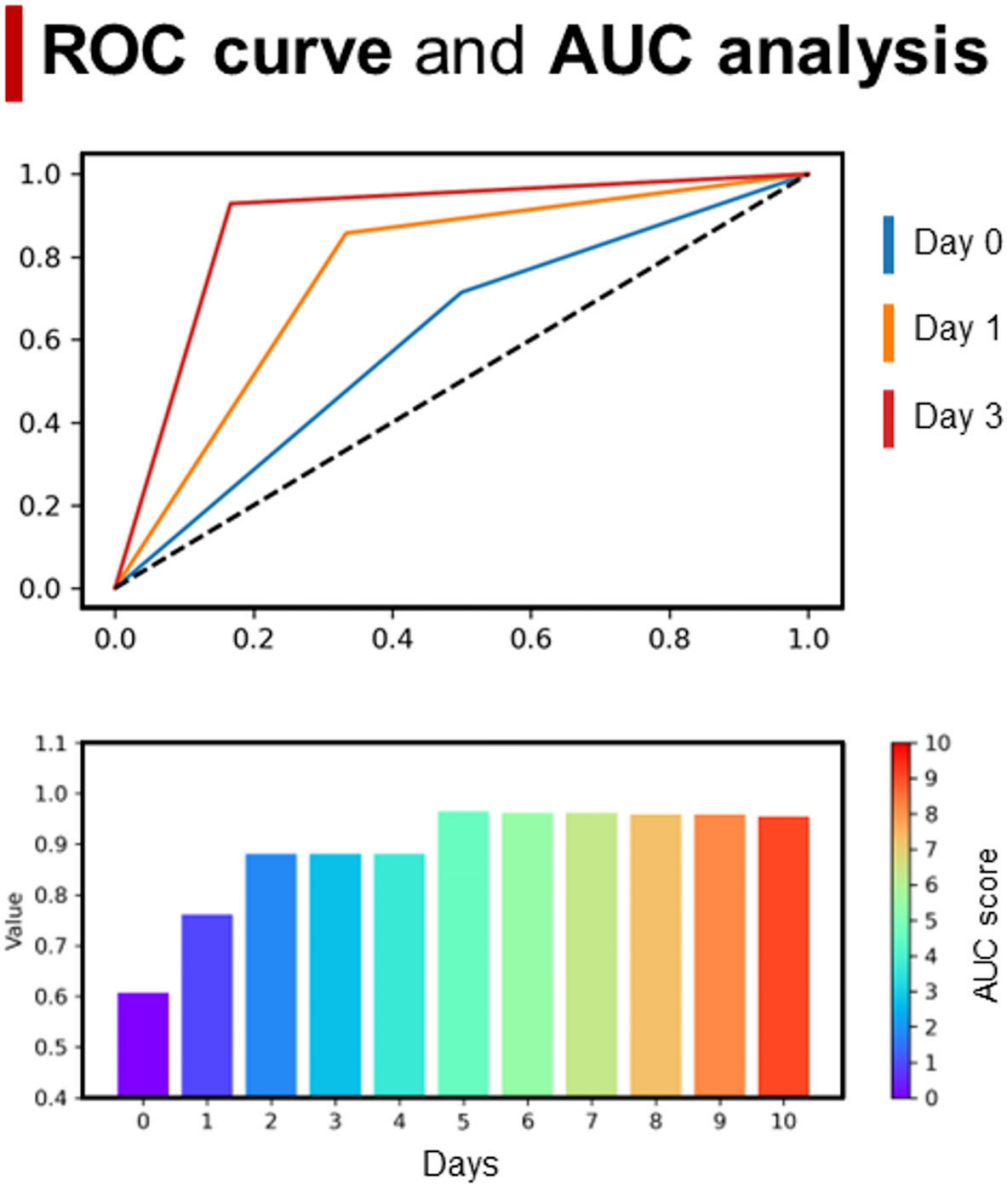
ROC curve and AUC analysis. Top panel: ROC curve for PS‐isochoria logistic regression model for Day 0, Day 1, and Day 3. Bottom panel: AUC score for the full days considered using PS‐isochoria as predictor outcome in the logistic regression.

Finally, a post‐hoc analysis was performed to investigate ROC and AUC scores using the first model (PS‐isochoria), considering its best results in terms of accuracy, BIC, and significance. As observed in Figure 5, this analysis showed great accuracy in discriminating between favorable and unfavorable outcomes already after 3 days and after 5 days, with a high level of stability.

## Discussion

Pupils' size and reactivity are part of the clinical evaluation of coma patients.^1^ They can provide early detection of neuro‐worsening and life‐threatening events (e.g., raise in ICP, transtentorial erniation) at the bedside of unresponsive/non‐communicative patients. Moreover, pupillary indices (mainly photo‐reactivity) on admission, together with other clinical data such as the GCS, are part of the predictive scores used in acute TBI coma patients to estimate long‐term outcome.^2^, ^3^, ^4^ However, long‐term prediction of outcome remains a highly relevant and clinically important issue.^8^

There has been recent interest in the dynamics of physiological patterns and variations over time of bio signals in complex biological systems, with possible applications also in critically ill patients.^8^, ^9^, ^10^, ^11^

In the present study, we were interested in applying this novel time‐based perspective for tracking pupillary dynamics (size, reactivity, and their changes over time) over an extended period of time in the ICU to determine if these pupillary dynamic measures predicted long‐term outcome in a group of acute TBI coma patients.

The main finding is that pupillary indices over time are informative in discriminating patients with favorable versus unfavorable outcomes in the first days in the Neuro‐ICU even under sedation condition.

In the first 10 days of ICU stay, patients with favorable outcome had more isochoric and photo‐reactive pupils, and these features were consistent and stable over time. On the contrary, patients with an unfavorable outcome had more variable measures that tended to stabilize over time toward pathological values (Fig. 2).

Moreover, in a model selection analysis, PS‐isochoria‐percentage if measured for at least 5 days after the Neuro‐ICU admission, was the most accurate in discriminating between outcomes when measured alone (BIC and accuracy) or in combination with other pupillary indices (p‐value) (Fig. 4).

Thus, the present study highlights the value of tracking pupillary indices over time in acute TBI coma patients to capture predictive information for outcome.^8^

In our cohort, all measures in the first few days were correlated with long‐term outcome, but the highest correlation value was reached overall around 5–6 days after ICU‐admission. In addition, in our cohort, the pupillary size over time was found to be the most powerful predictor long‐term outcome.

We suggest that tracking pupillary indices (both in terms of reactivity and size) not only for neuro‐monitoring but also for long‐term prediction is a promising and feasible application, especially considering the availability in the ICU of tools such as automated pupillometry that can provide quantitative and objective measures.^13^, ^14^, ^15^

This technique allows for more accurate measures, especially for the pupillary light reflex, which in our study may have been limited by manual and dichotomized evaluation.^14^

To‐date, we are aware of one study looking at time‐dependent trends of pupillary indices in TBI coma patients for correlation with episodes of raised ICP and the cumulative burden of such abnormalities on long‐term outcome.^12^ In our study, we did not correlate pupillary signals with ICP values^16^ since the neuro‐invasive monitoring was not required for all patients. To our knowledge, this is the first study that, first, tracks the dynamics of pupillary signals during the entire ICU stay of a group of acute TBI coma patients and, second, links these patterns with long‐term outcome, specifically the determination of the optimal time window of observation.

The study has several limitations. We cannot exclude the effects of medications on pupillary measures. However, there is still no robust data about the specific effects of medications on pupillary measures in ICU patients^13^, ^17^ Benzodiazepines can reduce the velocity of pupillary constriction, and catecholamines can increase the amplitude of the constriction.^18^ Propofol in isolation has been reported to reduce the pupil's diameter, though the impact on pupillary measures in the presence of other medications is still under investigation.^19^

Indeed, the overall treatment effects on pupillary measures are challenged by the simultaneous exposure to different medications, as is typical of ICU patients.

Nevertheless, in our cohort, we still observed significant differences in pupillary dynamics between outcome groups during a time window when these multiple medications were present and were known to affect the GCS evaluation.

Notably, the two groups were not statistically different in terms of clinical variables except for the higher prevalence of tSAH in the F group (Table 1).

Finally, our study is limited by a relatively small sample size and should thus be considered preliminary. However, the study is based on repeated within‐subject measures (an average of 191 data points per subject for each pupillary index) that provide a sensitive statistical design. Our findings are robust, showing the clinical relevance of dynamical pupillary measures for prognostication of outcome in TBI coma patients.^20^

Future studies with larger cohorts are needed and may consider taking into account a multi‐organ dynamic analysis (e.g., brain, body signals)^9^, ^10^, ^11^, ^21^ to catch the multi‐scale complexity of bio signals' interactions and their potential value for clinical prognosis and response to therapy.

## Conclusions

Time‐dependent tracking of pupillary dynamics is a promising application for ICU monitoring and long‐term prognosis in acute TBI coma patients.

## Author Contributions

E.M., C.F, A.S, M.M, M.C contributed to conception and design of the study. All authors contributed to acquisition, analysis of data, and drafting of the manuscript.

## Funding Information

Cariparo Foundation Excellence grant 2018 (Agreement N.55403); H2020‐MSCA‐ITN‐2019; Ministry of Health Italy (RF‐2008‐12366899).

## Conflicts of Interest

The authors report no conflicts of interest.

## Supporting information


Appendix S1

## Data Availability

Data and codes reported in the present study are available at https://github.com/CorbettaLab.
