# Supplementary material for: Pupillary dynamics predict long‐term outcome in a cohort of acute traumatic brain injury coma patients
Source: Ann Clin Transl Neurol. 2023 Aug 28;10(10):1854–62. doi: 10.1002/acn3.51879 (PMC10578890; doi:10.1002/acn3.51879)

**Supplementary Material**

**Table S1. Values of BIC, accuracy, and 1-p value in the prediction model.** Values of BIC, Accuracy, and 1-p value for each day and for all possible combinations of the 4 indexes are used as predictors (A= PS isochoria %; B= PS jumps rate; C=PR presence %; D=PR jumps rate).

BIC

|  | **A** | **B** | **C** | **D** | **A+B** | **A+C** | **A+D** | **B+C** | **B+D** | **D+C** | **A+B+C** | **A+B+D** | **A+C+D** | **B+C+D** | **A+B+C+D** |
| --- | --- | --- | --- | --- | --- | --- | --- | --- | --- | --- | --- | --- | --- | --- | --- |
| **0** | 25.06 | 30.43 | 27.73 | 30.43 | 28.05 | 27.96 | 28.05 | 30.73 | 33.42 | 30.73 | 30.96 | 31.05 | 30.96 | 33.73 | 33.96 |
| **1** | 23.08 | 27.61 | 27.89 | 30.43 | 24.90 | 25.24 | 26.08 | 29.55 | 30.60 | 30.89 | 27.51 | 27.89 | 28.24 | 32.55 | 30.50 |
| **2** | 18.12 | 27.56 | 25.06 | 27.89 | 20.45 | 19.87 | 20.37 | 28.04 | 30.40 | 28.06 | 22.86 | 23.31 | 22.86 | 31.04 | 25.86 |
| **3** | 15.47 | 25.82 | 23.34 | 26.87 | 17.77 | 17.31 | 17.82 | 26.31 | 28.77 | 24.43 | 20.31 | 20.69 | 19.56 | 27.43 | 22.56 |
| **4** | 14.51 | 25.91 | 23.63 | 26.73 | 15.84 | 15.84 | 16.28 | 26.45 | 28.85 | 26.46 | 18.71 | 18.81 | 18.83 | 29.43 | 21.51 |
| **5** | 13.56 | 24.20 | 22.12 | 25.41 | 14.30 | 14.82 | 15.24 | 25.04 | 27.05 | 24.75 | 17.29 | 16.79 | 17.75 | 27.74 | 19.78 |
| **6** | 12.10 | 24.03 | 21.48 | 24.93 | 14.02 | 13.56 | 13.86 | 24.43 | 26.78 | 24.16 | 16.27 | 16.76 | 16.43 | 27.00 | 18.95 |
| **7** | 5.67 | 20.42 | 20.96 | 20.09 | 8.50 | 8.50 | 8.50 | 23.23 | 22.90 | 22.91 | 11.33 | 11.33 | 11.33 | 25.73 | 14.17 |
| **8** | 5.28 | 14.51 | 15.86 | 13.45 | 7.92 | 7.92 | 7.92 | 17.11 | 15.51 | 14.62 | 10.56 | 10.56 | 10.56 | 16.58 | 13.20 |
| **9** | 5.28 | 14.22 | 15.86 | 13.45 | 7.92 | 7.92 | 7.92 | 16.78 | 15.80 | 14.62 | 10.56 | 10.56 | 10.56 | 16.58 | 13.20 |
| **10** | 5.13 | 13.95 | 15.50 | 13.17 | 7.69 | 7.69 | 7.69 | 16.43 | 15.41 | 14.20 | 10.26 | 10.26 | 10.26 | 16.00 | 12.82 |

Accuracy

|  | **A** | **B** | **C** | **D** | **A+B** | **A+C** | **A+D** | **B+C** | **B+D** | **D+C** | **A+B+C** | **A+B+D** | **A+C+D** | **B+C+D** | **A+B+C+D** |
| --- | --- | --- | --- | --- | --- | --- | --- | --- | --- | --- | --- | --- | --- | --- | --- |
| **0** | 65.00 | 70.00 | 75.00 | 70.00 | 65.00 | 65.00 | 65.00 | 75.00 | 70.00 | 75.00 | 65.00 | 65.00 | 65.00 | 75.00 | 65.00 |
| **1** | 80.00 | 75.00 | 70.00 | 70.00 | 65.00 | 80.00 | 80.00 | 70.00 | 75.00 | 70.00 | 65.00 | 65.00 | 80.00 | 70.00 | 65.00 |
| **2** | 90.00 | 70.00 | 80.00 | 70.00 | 85.00 | 90.00 | 90.00 | 75.00 | 70.00 | 75.00 | 85.00 | 85.00 | 90.00 | 70.00 | 85.00 |
| **3** | 90.00 | 70.00 | 80.00 | 75.00 | 85.00 | 85.00 | 85.00 | 75.00 | 70.00 | 75.00 | 80.00 | 80.00 | 80.00 | 70.00 | 75.00 |
| **4** | 90.00 | 70.00 | 80.00 | 75.00 | 90.00 | 90.00 | 90.00 | 75.00 | 65.00 | 75.00 | 90.00 | 80.00 | 80.00 | 65.00 | 75.00 |
| **5** | 95.00 | 75.00 | 80.00 | 70.00 | 85.00 | 90.00 | 90.00 | 75.00 | 65.00 | 75.00 | 80.00 | 75.00 | 85.00 | 70.00 | 75.00 |
| **6** | 94.74 | 73.68 | 78.95 | 68.42 | 89.47 | 89.47 | 89.47 | 78.95 | 63.16 | 78.95 | 84.21 | 84.21 | 84.21 | 73.68 | 78.95 |
| **7** | 94.12 | 76.47 | 76.47 | 76.47 | 88.24 | 88.24 | 88.24 | 64.71 | 70.59 | 70.59 | 82.35 | 76.47 | 88.24 | 64.71 | 70.59 |
| **8** | 92.86 | 85.71 | 78.57 | 85.71 | 78.57 | 92.86 | 85.71 | 71.43 | 78.57 | 85.71 | 71.43 | 85.71 | 85.71 | 92.86 | 78.57 |
| **9** | 92.86 | 85.71 | 78.57 | 85.71 | 78.57 | 92.86 | 85.71 | 78.57 | 78.57 | 85.71 | 71.43 | 78.57 | 85.71 | 92.86 | 85.71 |
| **10** | 92.31 | 84.62 | 76.92 | 84.62 | 76.92 | 92.31 | 84.62 | 76.92 | 76.92 | 84.62 | 69.23 | 76.92 | 84.62 | 92.31 | 92.31 |

1 – p value

|  | **A** | **B** | **C** | **D** | **A+B** | **A+C** | **A+D** | **B+C** | **B+D** | **D+C** | **A+B+C** | **A+B+D** | **A+C+D** | **B+C+D** | **A+B+C+D** |
| --- | --- | --- | --- | --- | --- | --- | --- | --- | --- | --- | --- | --- | --- | --- | --- |
| **0** | 0.06 | NaN | 0.13 | NaN | NaN | 0.15 | NaN | NaN | NaN | NaN | NaN | NaN | NaN | NaN | NaN |
| **1** | 0.03 | 0.17 | 1.00 | NaN | 0.10 | 0.14 | NaN | 0.57 | NaN | NaN | 0.25 | NaN | NaN | NaN | NaN |
| **2** | 0.02 | 0.22 | 1.00 | 1.00 | 0.06 | 0.10 | 0.07 | 0.99 | 0.78 | 1.00 | 0.19 | 0.15 | 0.19 | 1.00 | 0.30 |
| **3** | 0.03 | 0.09 | 0.17 | 0.15 | 0.14 | 0.13 | 0.10 | 0.39 | 0.25 | 1.00 | 0.24 | 0.25 | 0.29 | 1.00 | 0.42 |
| **4** | 0.03 | 0.10 | 0.12 | 0.14 | 0.23 | 0.15 | 0.14 | 0.28 | 0.27 | 0.26 | 0.37 | 0.42 | 0.28 | 0.44 | 0.61 |
| **5** | 0.04 | 0.08 | 0.22 | 0.11 | 0.43 | 0.21 | 0.18 | 0.42 | 0.23 | 0.29 | 0.63 | 0.90 | 0.36 | 0.45 | 0.96 |
| **6** | 0.13 | 0.10 | 0.34 | 0.11 | 0.36 | 0.38 | 0.37 | 0.61 | 0.26 | 0.39 | 0.55 | 0.56 | 0.56 | 0.56 | 0.64 |
| **7** | 1.00 | 0.20 | 0.57 | 0.14 | 1.00 | 1.00 | 1.00 | 0.51 | 0.35 | 0.35 | 0.00 | 1.00 | 1.00 | 0.54 | 1.00 |
| **8** | 1.00 | 0.18 | 0.35 | 0.16 | 1.00 | 1.00 | 1.00 | 0.39 | 0.34 | 1.00 | 1.00 | 1.00 | 1.00 | 1.00 | 1.00 |
| **9** | 1.00 | 0.17 | 0.35 | 0.16 | 1.00 | 1.00 | 1.00 | 0.37 | 0.34 | 1.00 | 1.00 | 1.00 | 1.00 | 1.00 | 1.00 |
| **10** | 1.00 | 0.19 | 0.38 | 0.17 | 1.00 | 1.00 | 1.00 | 0.40 | 0.35 | 1.00 | 1.00 | 1.00 | 1.00 | 1.00 | 1.00 |

**Figure S1: ICP in the two outcome groups.** Comparison of the ICP distribution between the two outcome groups (F in green; U in blue) through the non-parametric Mann-whitney test.


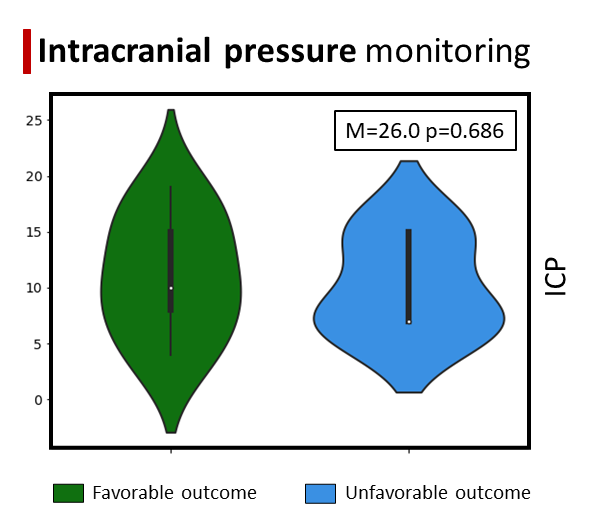

Supplement: Supplementary file 1 — Appendix S1 [file ACN3-10-1854-s001.docx]
